# Predicting Clinically Significant Improvement After Robot-Assisted Upper Limb Rehabilitation in Subacute and Chronic Stroke

**DOI:** 10.3389/fneur.2021.668923

**Published:** 2021-07-01

**Authors:** Jae Joon Lee, Joon-Ho Shin

**Affiliations:** ^1^Department of Rehabilitation Medicine, National Rehabilitation Center, Ministry of Health and Welfare, Seoul, South Korea; ^2^Translational Research Center for Rehabilitation Robots, National Rehabilitation Center, Ministry of Health and Welfare, Seoul, South Korea

**Keywords:** robotics, upper extremity, minimal clinically important difference, prognosis, rehabilitation, stroke

## Abstract

Prior studies examining predictors of favorable clinical outcomes after upper limb robot-assisted therapy (RT) have many shortcomings. Therefore, the aim of this study was to identify meaningful predictors and a prediction model for clinically significant motor improvement in upper limb impairment after RT for each stroke phase. This retrospective, single-center study enrolled patients with stroke who received RT using InMotion2 along with conventional therapy (CT) from January 2015 to September 2019. Demographic characteristics, clinical measures, and robotic kinematic measures were evaluated. The primary outcome measure was the Fugl-Meyer Assessment-Upper Extremity (FMA-UE) and we classified patients with improvement more than the minimal clinically important difference as responders for each stroke phase. Univariable and multivariable logistic regression analyses were performed to assess the relationship between potential predictors and RT responders and determine meaningful predictors. Subsequently, meaningful predictors were included in the final prediction model. One hundred forty-four patients were enrolled. The Hand Movement Scale and time since onset were significant predictors of clinically significant improvement in upper limb impairment (*P* = 0.045 and 0.043, respectively), as represented by the FMA-UE score after RT along with CT, in patients with subacute stroke. These variables were also meaningful predictors with borderline statistical significance in patients with chronic stroke (*P* = 0.076 and 0.066, respectively). Better hand movement and a shorter time since onset can be used as realistic predictors of clinically significant motor improvement in upper limb impairment after RT with InMotion2 alongside CT in patients with subacute and chronic stroke. This information may help healthcare professionals discern optimal patients for RT and accurately inform patients and caregivers about outcomes of RT.

## Introduction

Upper extremity dysfunction commonly occurs after a stroke, affecting ~80% of people with acute stroke and 50% of people with chronic stroke. It negatively affects activities of daily living as well as social activities (1, 2). Therefore, improving upper extremity function is a primary therapeutic goal in stroke rehabilitation (3). Several systematic reviews suggest that repetitive, task-specific, and intensive therapy may result in motor improvement after stroke (4, 5). Robotic systems can provide more consistent, intensive, and repetitive training without fatigue, along with task-specific training by easily applying new constraints to optimize the required movement pattern, as compared to conventional therapy (CT) (6). Recent systematic reviews on robot-assisted therapy (RT) of the upper limb after stroke have reported that a more meaningful clinical outcome is obtained with RT than with CT (7, 8).

Identifying the predictors of a favorable clinical outcome after RT is imperative. It could help healthcare professionals to identify those patients who are best suited for RT and to accurately guide patients and caregivers about the outcomes of RT. It would also improve the cost efficiency of RT, which is currently steep.

Several studies have been conducted to identify predictors so that favorable outcomes with upper limb RT can be ensured among patients with stroke. Hsieh et al. (9) enrolled 55 patients with stroke who had undergone RT using the Bi-Manu-Track (Reha-Stim, Berlin, Germany) and found that the Box and Block Test score and female sex could predict favorable outcomes in the Fugl-Meyer Assessment-Upper Extremity (FMA-UE) and Motor Activity Log scores. The same researchers conducted a secondary analysis by enrolling 66 patients with stroke using the cohort data generated in the aforementioned study (10). Spasticity of the upper extremity and kinematic measures were added to the potential predictors analyzed in the previous study, and lessened flexor synergy and spasticity were found to be predictors of a favorable Wolf Motor Function Test result. Franceschini et al. (11) demonstrated that the Box and Block Test score, FMA-UE score, and Motricity Index (MI) upper limb could predict a favorable post-RT Modified Barthel Index using data from 60 patients with stroke who had undergone RT using InMotion2 (Interactive Motion Technologies, Watertown, MA, USA). Duret et al. (12) enrolled 46 patients with stroke who had undergone RT using InMotion2 and demonstrated that the time since onset and Fugl-Meyer Assessment (FMA) shoulder/elbow score were predictors of a favorable post-RT FMA shoulder/elbow score. Although these two variables could predict improvement after RT, they could not predict improvement more than the minimal clinically important difference (MCID), which is the minimal effect that has clinical relevance in patient management (13).

Many studies have been conducted to identify predictors; however, they had several limitations, such as inconsistently identified predictors, inadequate number of subjects, limited numbers of analyzed potential predictors, and unclearly distinguished stroke phases, despite the difference in recovery depending on the stroke phase. No predictor identified thus far can predict an improvement more than the MCID in the FMA-UE, the main tool used to assess impairment. We hypothesized that some predictors may have the potential to predict improvement more than the MCID of FMA-UE after RT, and these predictors may vary depending on the phase of the stroke. Therefore, the aim of the present study was to identify meaningful predictors and a prediction model for clinically significant motor improvement in cases of upper limb impairment after RT for each stroke phase.

## Methods

### Study Design and Setting

This retrospective, single-center study followed the Strengthening the Reporting of Observational Studies in Epidemiology guidelines (14). The Ethics Committee of the Institutional Review Board of the National Rehabilitation Center in South Korea approved this study (approval number, NRC-2019-04-030) and waived the requirement for informed consent because of the retrospective design.

From January 2015 to September 2019, patients with stroke who were admitted to the National Rehabilitation Center in South Korea, and who received RT using InMotion2, were enrolled in this study. The inclusion criteria were a definite diagnosis of unilateral stroke, as evidenced by computed tomography, magnetic resonance imaging, or medical records; a time since onset of ≥7 days for a first-ever stroke; and age ≥19 years. The exclusion criteria were as follows: neurological disorders other than stroke that can cause motor deficits, e.g., Parkinson disease, spinal cord injury, Guillain-Barré syndrome, traumatic brain injury, brain tumor, hypoxic brain injury, cerebral palsy, and peripheral neuropathy; spasticity in the elbow joint with a Modified Ashworth Scale (MAS) grade >3; severe upper extremity pain that could interfere with RT (Numeric Rating Scale score ≥5); upper extremity fracture within 3 months; uncontrolled severe medical conditions; a history of non-invasive brain stimulation; RT for <20 sessions; and incomplete medical records.

The patients' data were sourced from the electronic medical records in the database of our health care institute. The demographic, clinical, and robotic records were extracted. The patients' records were de-identified before analysis. The principal investigator (JH) conceived and designed the study, and an occupational therapist (SY) collected the data. Investigators (JJ and JH) performed data curation and statistical analysis and wrote and edited the paper.

We analyzed patients with stroke according to time since onset, which was classified as subacute phase (time since onset of ≥7 and <180 days) and chronic phase (time since onset of ≥180 days) (15).

### Intervention and Apparatus

Each patient participated in a total of 20 sessions of RT using InMotion2; patients underwent one 30-min RT session per day, 5 days a week for 4 weeks. InMotion2, which has been proven efficient and safe for patients with subacute and chronic stroke (16, 17), is a two-degrees-of-freedom end-effector type robotic device that provides shoulder-elbow flexion/extension training in the horizontal plane. In the seated position with the trunk restrained by a five-point seatbelt to minimize compensatory movement and with the forearm supported by a forearm cradle, each patient performed goal-directed reaching movements in the gravity-compensated horizontal plane. The patients were instructed to move the handle from the center target to each of eight peripheral targets positioned 45 degrees apart in circular arrangements, and the position of the handle was marked on the screen for real-time visual feedback. All the patients also received CT, according to the standardized rehabilitative protocol, involving range of motion exercises, strengthening exercises for the affected upper extremity, and activities of daily living training.

### Potential Predictors

To identify meaningful predictors, we included variables known to be related to outcome after therapeutic intervention (18, 19) and those suspected of clinical relevance, but not yet confirmed. Demographic characteristics [age, sex, time since onset, stroke subtype, stroke lesion (cortical, subcortical, or combined cortical and subcortical), and hemiplegic side], clinical measures [FMA-UE score, MI, Medical Research Council Scale for Muscle Strength (MRC) score, MAS grade at the elbow flexor muscle of the hemiplegic side, Hand Movement Scale (HMS), and Brunnstrom Recovery Stage (BRS)], and robotic kinematic measures [smoothness, reach error (RE), path error (PE), and independence] were selected for analysis.

The assessments of FMA-UE, MI, MAS, smoothness, RE, PE, and independence were conducted by experienced occupational therapists before the first RT session and after the last session. The evaluations of MRC-shoulder flexion, extension, abduction, and adduction; MRC-elbow flexion and extension; MRC-wrist flexion and extension; MRC-finger flexion and extension; HMS; and BRS were performed at admission.

#### Clinical Measures

The FMA-UE is a quantitative measure of motor impairment following a stroke and consists of 33 items rated on a three-point scale (maximum score, 66), with higher scores indicating less severe impairment (20). The scale is composed of sub-scores: 36 for the shoulder/elbow (FMA-A), 10 for the wrist (FMA-B), 14 for the hand (FMA-C), and 6 for coordination (FMA-D). These can be distributed into sub-scores of 42 for the proximal unit of the shoulder/elbow and coordination (FMA-Prox) and 24 for the distal unit of the wrist and hand (FMA-Dist).

The MI is based on the ability to move the upper extremity segment through a range of motion and to resist the force. The MI-upper limb consists of three domains (pinch grasp, elbow flexion, and shoulder abduction). Each domain is scored between 0 and 33, and the total upper limb score (maximum score, 100) is calculated by adding one to the sum of the three domain scores (21).

The MRC score ranges from 0 to 5, with higher scores representing greater muscle strength (22). The MRC-upper extremity score was calculated by summing the MRC-shoulder, MRC-elbow, MRC-wrist, and MRC-finger scores, whereas the MRC-shoulder score was calculated by adding the MRC-shoulder flexion, extension, abduction, and adduction scores. The MRC-elbow, MRC-wrist, and MRC-finger scores were each calculated as the sum of the MRC-elbow flexion and extension, MRC-wrist flexion and extension, and MRC-finger flexion and extension scores, respectively.

The MAS measures spasticity, with a higher grade indicating higher spasticity (23). The MAS spasticity grades of 1+, 2, 3, and 4 were converted to 2, 3, 4, and 5, respectively, while grade 1 remained the same.

The HMS ranges from 1 to 6 and evaluates the ability to perform hand movements of different degrees of difficulty, with a higher number representing better hand movement (24).

The BRS ranges from 1 to 6 and describes the stereotypical stages of motor recovery, starting with flaccidity to full recovery of motor function (25). The BRS consists of different parts; the two parts concerning the upper arm (BRS-upper arm) and the hand (BRS-hand) were used herein.

#### Robotic Kinematic Measures

Robotic kinematic measures (e.g., smoothness, RE, PE, and independence) were used as potential predictors. Assessments of kinematic measures consist of point-to-point reaching movements and circle drawing movements (26). The point-to-point reaching movement assessment was used to calculate smoothness, RE, and PE, while the circle drawing assessment was conducted to calculate independence. Smoothness was calculated as the mean of the speed divided by the peak speed and is expressed as a value ranging from 0 to 1, where a value closer to 1 indicates better control of movement speed (27). RE and PE represent the ability to move accurately along a straight path toward the center of targets and toward targets, respectively. RE was calculated as the normalized summed difference of the end of the reach from the center of the target with respect to time. PE was calculated as the normalization of the summed deviations from the desired straight path and the participant's actual path from one point to another with respect to time. RE and PE are expressed as a value ranging from 0 to 1, with a value closer to 0 indicating better performance (28). Independence was calculated as the ratio between the major and minor axes of the ellipse that best represents the path drawn by the hand during the circle drawing assessment. Values range from 0 to 1, where values closer to 1 represent fitting ellipses that are closer to a circle, and indicate better coordination of shoulder and elbow movements (29).

#### Outcome Measure

Since RT focuses upon upper limb impairment, we chose the FMA-UE as the primary outcome measure and calculated the difference in the FMA-UE score before and after RT (ΔFMA-UE). We considered 9 and 5.25 as the MCID for patients with subacute and chronic stroke, respectively (30, 31). As such, patients with subacute stroke who had a ΔFMA-UE value ≥9 and patients with chronic stroke who had a ΔFMA-UE value ≥5.25 were classified as responders in this study. Those with values below the aforementioned were classified as non-responders.

### Statistical Analysis

The sample size calculation estimated that 58 subjects would provide 80% power with 5% α and an odds ratio of 2.5 (power analysis using logistic regression according to the guidelines of Lipsey & Wilson and G Power 3.1.9.7 software) (32).

Continuous variables are presented as means and standard deviations, and categorical variables are presented as numbers and percentages. The normal distribution of continuous variables was assessed using the Kolmogorov-Smirnov test. Meaningful predictors were determined using univariable and multivariable logistic regression analyses (33). We performed univariable logistic regression analyses to assess the relationship between potential predictors and the outcome measure, and extracted variables for which the *P*-value was <0.25 (34). These variables were further tested for correlations among variables using the Pearson or Spearman correlation test depending on the distribution (normal or not). We excluded variables that had a high correlation (|R| > 0.7) (35) and a low odds ratio. To prevent overfitting, we calculated outcome events per predictor variable (EPV) using the number of selected variables. It is recommended that the EPV should be at least 10:1 (36). Next, multivariable stepwise logistic regression analysis was used to determine meaningful predictors. Subsequently, meaningful predictors with a significance level of <0.05 were included in the final prediction model. The goodness-of-fit of the final model and each meaningful predictor was tested with the Hosmer-Lemeshow test. Finally, receiver operating characteristic curves were used to assess the predictive capacity of the developed prediction model and to determine the most reliable cut-off score of each meaningful predictor in relation to responders of RT. Herein, 95% confidence intervals (CIs) are reported for the area under the receiver operating characteristic curves (AUCs). A *P*-value < 0.05 was considered reflective of statistical significance. Statistical analyses were conducted using the IBM SPSS Statistics for Windows, version 20.0 (IBM Corp., Armonk, NY, USA).

## Results

### Patient Characteristics

**Figure 1:**
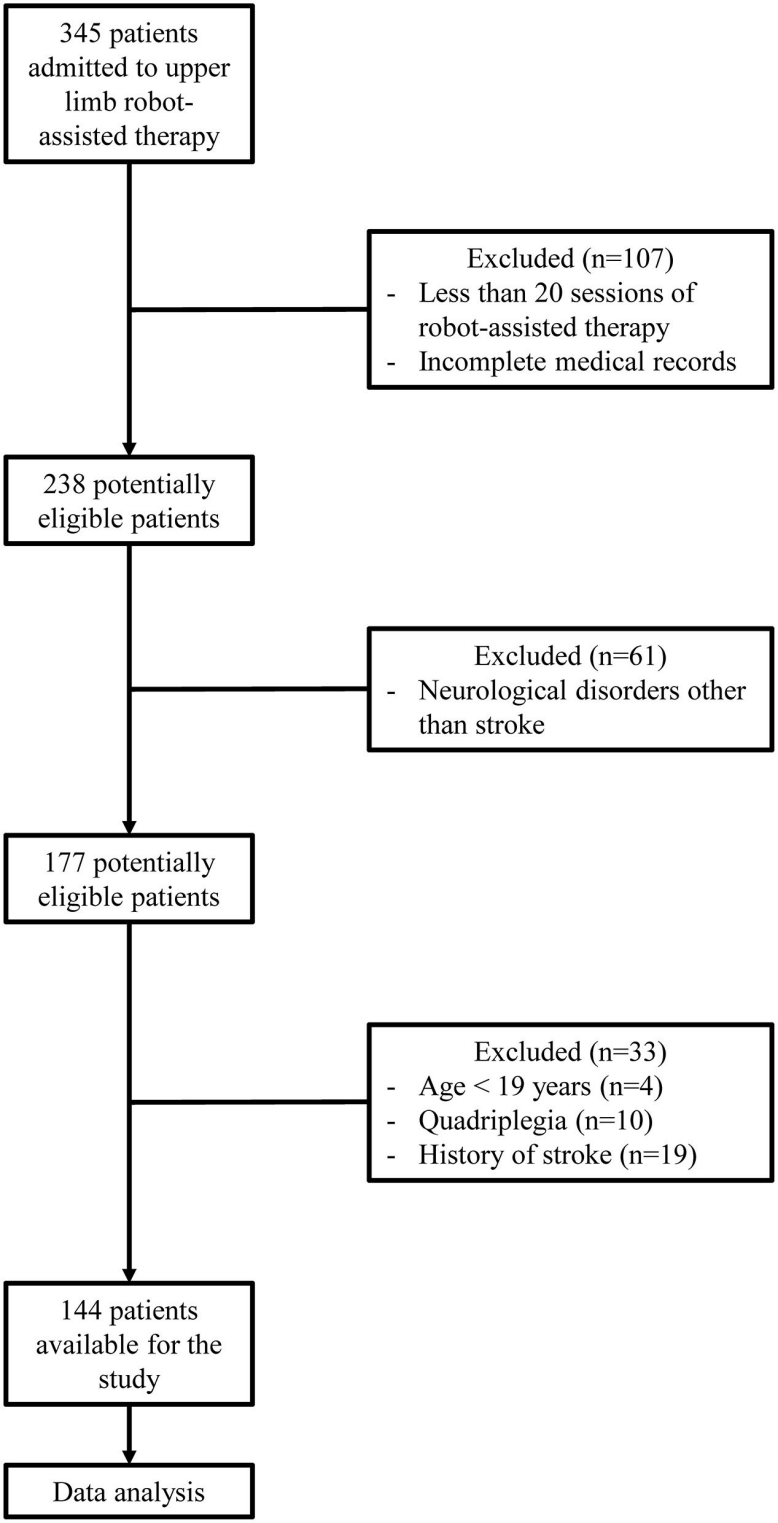
Flow diagram of the selection procedures of stroke patients.

Three hundred forty-five patients underwent RT using InMotion2 between January 2015 and September 2019. Among them, 107 were excluded because of termination of the RT due to medical abnormalities, pain, decreased patient motivation, unexpected discharge, or the absence of evaluation following RT and incomplete medical records. Upon exclusion of 61 patients who underwent RT for a diagnosis other than stroke, 4 patients who were <19 years old, 10 quadriplegic patients, and 19 patients who had a history of stroke, a total of 144 patients were enrolled (Figure 1).

Overall, there were 86 patients with subacute stroke and 58 with chronic stroke. Among patients with subacute stroke, there were 25 responders and 61 non-responders. Among those with chronic stroke, there were 12 responders and 46 non-responders. The characteristics of responders and non-responders by stroke phase are shown in Table 1.

**Table 1:** Baseline characteristics of responders and non-responders according to stroke phase.

	**Subacute phase**		**Chronic phase**	
**Characteristics**	**Responder (*n* = 25)**	**Non-responder (*n* = 61)**	**Responder (*n* = 12)**	**Non-responder (*n* = 46)**
**Demographic characteristic**				
Age, years	56.3 (12.2)	54.7 (14.0)	57.3 (13.6)	56.0 (12.9)
Sex, male	19 (76.0)	38 (62.3)	10 (83.3)	35 (76.1)
Female	6 (24.0)	23 (37.7)	2 (16.7)	11 (23.9)
Time since onset, days	91.8 (44.0)	110.1 (34.0)	280.3 (64.9)	417.3 (228.4)
Stroke subtype, ischemic	14 (56.0)	30 (49.2)	4 (33.3)	23 (50)
Lesion, cortical	1 (4.0)	3 (5.0)	0 (0.0)	1 (2.1)
Subcortical	19 (76.0)	40 (65.5)	10 (83.3)	30 (65.2)
Combined	5 (20.0)	18 (29.5)	2 (16.7)	15 (32.7)
Hemiplegic side, right	14 (56.0)	26 (42.6)	5 (41.7)	22 (47.8)
**Clinical measure**				
FMA-UE	20.5 (7.9)	20.4 (8.7)	20.2 (9.5)	18.9 (9.3)
MAS	0.9 (0.9)	1.1 (0.7)	1.3 (0.78)	1.5 (0.8)
HMS	2.6 (1.3)	2.1 (0.9)	3.0 (1.8)	2.2 (0.7)
**Robotic kinematic measure**				
Smoothness	0.425 (0.072)	0.421 (0.070)	0.460 (0.063)	0.440 (0.068)
Reach error	0.064 (0.058)	0.061 (0.0445)	0.062 (0.060)	0.062 (0.052)
Path error	0.031 (0.025)	0.027 (0.017)	0.026 (0.026)	0.028 (0.023)
Independence	0.548 (0.179)	0.526 (0.167)	0.538 (0.134)	0.538 (0.188)

*Values are the mean (standard deviation) for continuous data, and the number (percentage) for categorical data*.

*FMA-UE, Fugl-Meyer Assessment-Upper Extremity; MAS, Modified Ashworth Scale; HMS, Hand Movement Scale*.

### Potential and Meaningful Predictors

#### Subacute Phase

Variables identified through univariable logistic regression analysis of the relationship between potential predictors and responders of RT with a *P*-value < 0.25 were sex; time since onset; FMA-C score; MRC-wrist flexion, MRC-wrist extension, MRC-finger extension, and MRC-wrist scores; MAS grade; HMS; and BRS-hand (Supplementary Table 1). Among these variables, a high correlation was demonstrated between MRC-wrist flexion and MRC-wrist extension scores, MRC-wrist flexion and MRC-finger extension scores, MRC-wrist flexion and MRC-wrist scores, MRC-wrist extension and MRC-finger extension scores, MRC-wrist extension and MRC-wrist scores, and between the MRC-finger extension score and the HMS. We excluded the MRC-wrist flexion and MRC-wrist scores that had a low odds ratio. However, if an MRC-finger extension score had a low odds ratio, it was not excluded, as it was presumed to be a major potential predictor. Eight potential predictors were selected and the EPV was >10 (EPV = 10.75). Multivariable stepwise logistic regression analysis of selected potential predictors followed by application of a backward elimination procedure revealed the time since onset and HMS as significantly meaningful predictors (Table 2).

**Table 2:** Multivariable analyses using the MCID of the FMA-UE as the outcome measure according to stroke phase.

	**Subacute phase**					**Chronic phase**				
**Baseline characteristics**	**Unstandardized coefficient**	**Odds ratio**	**95% CI**		***P-*value**	**Unstandardized coefficient**	**Odds ratio**	**95% CI**		***P-*value**
Time since onset	−0.014	0.99	0.97	1.00	0.043^*^	−0.008	0.99	0.98	1.00	0.066^†^
HMS	0.451	1.57	1.01	2.44	0.045^*^	0.497	1.65	0.95	2.85	0.076^†^
Constant	−0.520	0.60			0.543	−0.078	0.93			0.958

*MCID, minimal clinically important difference; FMA-UE, Fugl-Meyer Assessment-Upper Extremity; CI, confidence interval; HMS, Hand Movement Scale*.

* *P < 0.05*;

† *P < 0.1 in the multivariable analysis*.

#### Chronic Phase

In the univariable logistic regression analysis of the relationship between potential predictors and responders of RT, the variables with a *P*-value < 0.25 were time since onset; MI-upper limb, MRC-wrist extension, MRC-finger flexion, MRC-finger extension, and MRC-finger scores; HMS; and BRS-hand (Supplementary Table 1). Among these variables, a high correlation was demonstrated between the MRC-finger flexion and MRC-finger scores, and between the MRC-finger extension and MRC-finger scores. The MRC-finger scores that had a low odds ratio were excluded. Seven potential predictors were finally selected. The EPV was <10, but was in line with the recommended range of ≥5–9 EPV (EPV = 8.3) (37). Multivariable stepwise logistic regression was conducted on the selected potential predictors, and the time since onset and HMS were identified as meaningful predictors using the backward elimination procedure (Table 2).

### Final Prediction Model and Meaningful Predictor Cut-Off Score

In the final prediction model, the time since onset and HMS were included in each subacute and chronic stroke model. Below are the final logistic regression equations.

$$\begin{array}{l} Subacute\;phase:\;Logit\;P\;\left(\Delta FMA-UE\;\geq 9\right)\\ \;=\;-0.520\;-\;0.014\;\times \;\left(time\;since\;onset\right)\;+\;0.451\;\times \;\left(HMS\right)\\ Chronic\;phase:\;Logit\;P\;\left(\Delta FMA-UE\;\geq 5.25\right)\\ \;=\;-0.078\;-\;0.008\;\times \;\left(time\;since\;onset\right)\;+\;0.497\;\times \;\left(HMS\right) \end{array}$$

**Figure 2:**
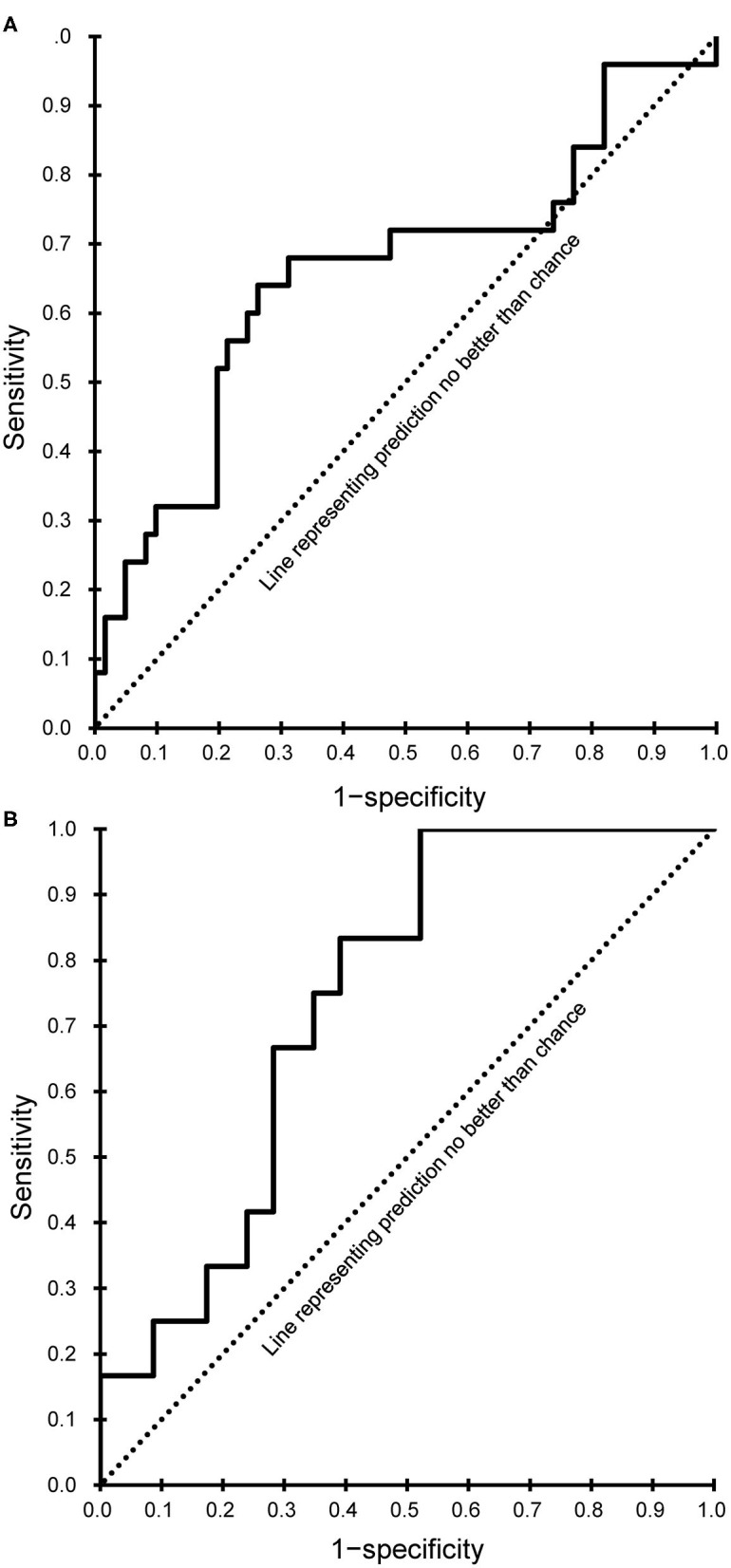
Receiver operating characteristic curves of the final prediction model. **(A)** Subacute phase. **(B)** Chronic phase.

Both models showed a good fit (Hosmer-Lemeshow test, *P* > 0.05), and the corresponding AUC values were calculated and plotted as receiver operating characteristic curves (Figure 2). AUC values with 95% CIs were 0.658 (95% CI, 0.520–0.797) for the subacute phase model and 0.739 (95% CI, 0.606–0.872) for the chronic phase model.

**Figure 3:**
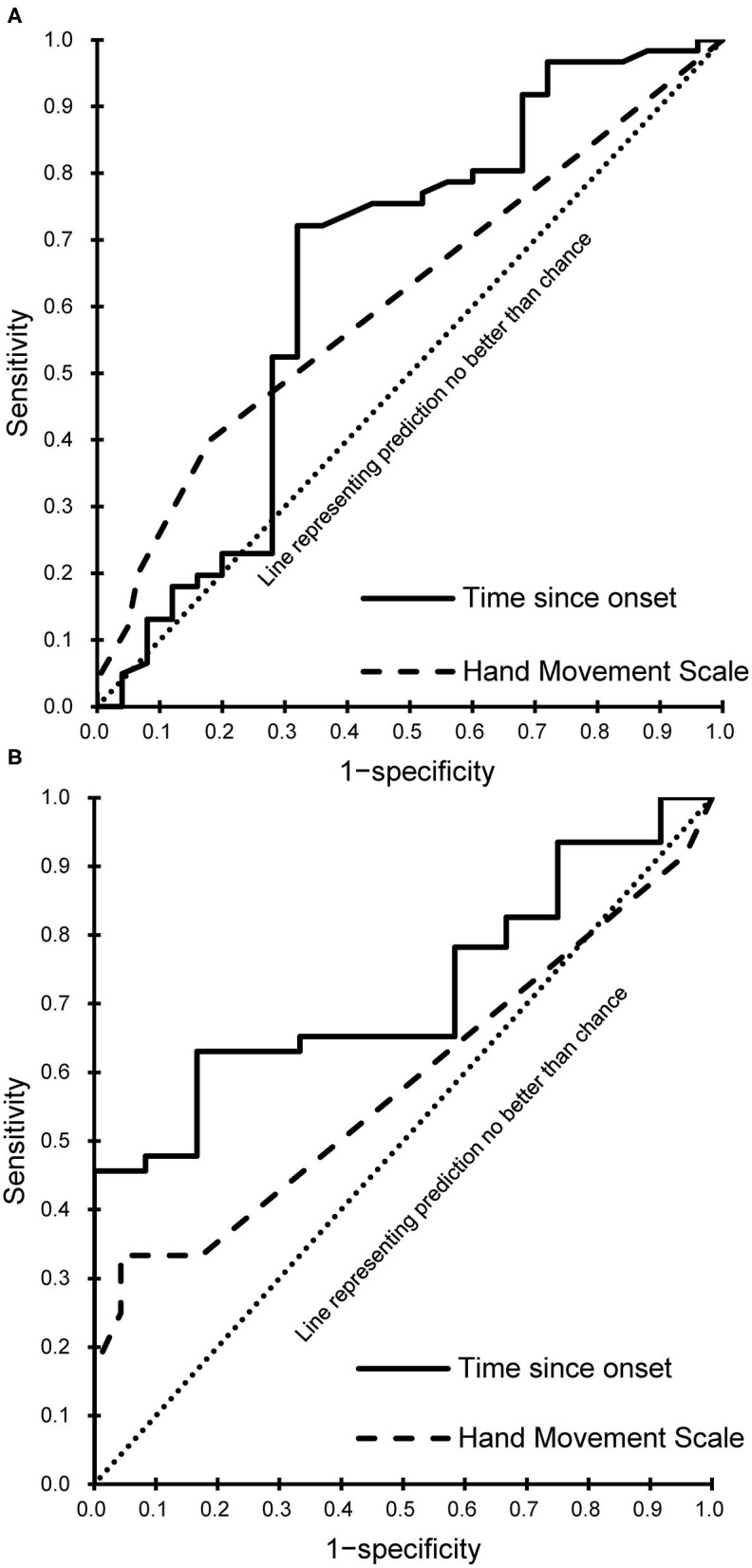
Receiver operating characteristic curves of the meaningful predictors. **(A)** Subacute phase. **(B)** Chronic phase.

Every meaningful predictor showed a good fit (Hosmer-Lemeshow test, *P* > 0.05). The sensitivity and specificity for the cut-off score of the meaningful predictors were calculated and plotted as receiver operating characteristic curves (Figure 3) for patients with subacute and chronic stroke. Corresponding AUC values with 95% CIs are shown in Table 3.

**Table 3:** The sensitivity and specificity for the cut-off score of the meaningful predictors according to stroke phase.

**Phase**	**Meaningful predictor**	**AUC**	**95% CI**		**Cut-off**	**Sensitivity**	**Specificity**
Subacute	Time since onset	0.65	0.50	0.79	97.5	68.9%	68%
	HMS	0.61	0.47	0.75	2.5	40%	82%
Chronic	Time since onset	0.72	0.58	0.86	299.5	65.2%	66.7%
	HMS	0.58	0.37	0.79	2.5	33%	82.6%

*AUC, area under the receiver operating characteristic curve; CI, confidence interval; HMS, Hand Movement Scale*.

## Discussion

This study demonstrated that the HMS and time since onset were significant predictors for clinically significant motor improvement in upper limb impairment, as represented by the FMA-UE score after RT with InMotion2 alongside CT in patients with subacute stroke. Similarly, the HMS and time since onset were meaningful predictors with borderline statistical significance in patients with chronic stroke.

This study demonstrated that the HMS is a meaningful predictor among patients with subacute and chronic stroke. A baseline HMS that exceeds 2.5, i.e., ≥3 was indicative of a favorable outcome post-RT. An HMS of 3 indicates possible active flexion and extension of all fingers in synergy. Active finger extension has been revealed as an indicator of better recovery of arm function in patients with stroke in multiple studies. Fritz et al. (38) confirmed that active finger extension could predict recovery following constraint-induced movement therapy. Additionally, Smania et al. (39) demonstrated that an MRC-finger extension score >3 could be a predictor of the subacute and chronic stroke phase recovery, and that an HMS >3 could predict recovery in the chronic phase; these results are supportive of the findings of our study. The HMS had a low sensitivity but a high specificity in the present study. Therefore, healthcare professionals can perform HMS when determining the beginning of RT in patients with a subacute or chronic stroke. In cases where the HMS score is ≤2, it can be explained to the patients or caregivers that it is difficult to expect the complete therapeutic effect of RT. This may lead to increased cost efficiency for RT, and the efficient use of hospital resources. The HMS is also easy to perform. For these reasons, the HMS is likely to be a suitable and convenient criterion for responders of RT.

The outcomes of this study are consistent with those of several prior studies showing that baseline dexterity is a major predictor of post-RT upper limb recovery. Hsieh et al. (9) and Huang et al. (10) demonstrated that the Box and Block Test score in patients with chronic stroke was a predictor of motor and functional outcomes following RT, whereas Franceschini et al. (11) confirmed that the Box and Block Test score was a predictor of post-RT functional outcome in patients with subacute stroke. Baseline hand movement, not baseline proximal upper limb function, predicts a favorable outcome; this may be explained by the fact that distal upper limb function is mostly represented unilaterally in the brain, whereas proximal upper limb function is represented bilaterally. Therefore, preservation of hand movement is more related to the degree of sparing of corticospinal pathways than it is to proximal upper limb function and represents a higher recovery potential (40, 41).

Herein, the time since onset was likewise identified as a meaningful predictor of a favorable outcome following RT. Undergoing RT at a shorter time since onset was more effective, specifically before 97.5 days since onset and 299.5 days since onset for patients with subacute and chronic stroke, respectively. Previous studies have confirmed that earlier intervention can predict favorable post-intervention outcomes in such cases. Duret et al. (12) and Mazzoleni et al. (42) suggested that early administration of RT could provide greater functional improvement. Paolucci et al. (18) demonstrated that CT was more effective in patients for whom it was initiated soon after stroke onset, compared to CT in those for whom it was initiated later. The predictive capability of the time since onset may not be surprising, because a shorter time after stroke may be associated with a greater potential for recovery, possibly improving the response to RT. Although stroke recovery is heterogeneous and the long-term effects of stroke are determined by the site and size of the initial stroke lesion, almost all stroke recovery follows a logarithmic pattern time course; in many stroke patients, motor recovery is almost complete after 8 to 12 weeks (1, 43). The time period of 97.5 days since onset that we identified is in line with the results of these prior studies. Moreover, the administration of RT within 299.5 days since onset in patients with chronic stroke was promising for significant recovery, albeit to a lesser degree than that observed for patients in the subacute phase. There is a growing body of evidence supporting the argument that the potential for neuroplasticity and adaptation continues and that motor function improves over time in chronic stroke (16, 44).

The MCID of the FMA-UE has been established 5.25 for patients with chronic stroke (31). For subacute stroke, we selected an FMA-UE score of 9 as the MCID (30). Although another study found an MCID of 4 for patients with subacute stroke (45), we chose 9 because motor recovery in the subacute phase is better than that in the chronic phase. Additionally, mean time since onset in our population was closer to that of Narayan Arya et al. (30) than that of Lundquist et al. (45).

Interestingly, the predictors found in responders of RT among patients with subacute and chronic stroke, were HMS and time since onset. Although both predictors were statistically significant for patients with subacute stroke, they had borderline statistical significance for patients with chronic stroke. This can be explained by combining the characteristics of the two variables. As demonstrated earlier, although a high HMS demonstrates a high potential for recovery due to relatively well-preserved corticospinal pathways following a stroke, RT may have not been as effective in the chronic phase as it was in the subacute phase, and other factors, such as muscle atrophy, fatigue, and pain, may have had a greater effect than the neural substrate related to neural plasticity.

No robotic kinematic measure examined in this study was able to predict responders of RT. This finding is supported by the study of Duret et al. (12), in which predictors of a favorable motor outcome in patients with subacute stroke were identified. However, robotic kinematic measures were unable to predict favorable post-RT outcomes because the measures currently being used are insufficient. Schwarz et al. (46) conducted a systematic review on the kinematic assessment of upper limb movements and demonstrated that the reliability, correlation with the FMA-UE score, and ability to detect longitudinal changes of the kinematic measures used were low. However, Krebs et al. (47) reported that a standard clinical outcome measure and significant correlation was observed when kinematic and kinetic measures were included simultaneously. As such, if an upgraded standardized kinematic measure or kinematic and kinetic measure is developed, additional research using it as a potential predictor may be needed.

This study has several limitations. First, as this was a retrospective study, potential confounding factors that could have affected the clinical outcomes were not accounted for, and because patients of just one rehabilitation hospital were studied, there may have been selection bias. Nonetheless, given that a relatively standardized rehabilitation therapy was conducted, and as all study subjects were patients admitted to the same rehabilitation hospital, environmental factors were minimized. Additionally, this study was conducted with a sufficient number of patients with subacute and chronic stroke. Second, it is difficult to say whether the identified predictors solely predicted favorable post-RT outcomes, as CT was administered along with RT. However, considering that CT and RT were administered to all patients and that RT is rarely administered without CT, the outcomes of this study can be used as realistic predictors. Third, early and late subacute phases were not divided despite the chances of the influence of these phases on recovery and outcome. Fourth, aside from the chronic phase prediction model and the time since onset in the chronic phase, the AUC values of the remaining prediction model and meaningful predictors were <0.7, indicating insufficient discrimination ability. Lastly, other kinematic measures such as movement duration, peak velocity and peak acceleration known to be related to outcome after RT (48), and neuropsychological impairments such as aphasia and neglect known to be related to post-stroke motor recovery (49, 50), psychosocial, and emotional factors, which may have affected the outcome, were not included as potential predictors. Therefore, controlled, prospective, and multicenter studies including a more comprehensive set of potential predictors are required to validate and improve our results in the future.

## Conclusions

Better hand movement and a shorter time since onset can realistically serve to predict clinically significant motor improvement in upper limb impairment after RT with InMotion2 alongside CT, in patients with subacute and chronic stroke, whereas other demographic characteristics and robotic kinematic measures cannot predict responders of RT. These findings may assist healthcare professionals in discerning optimal patients for RT and in accurately informing patients and caregivers about the outcomes of RT.

## Data Availability Statement

The raw data supporting the conclusions of this article will be made available by the authors, without undue reservation.

## Ethics Statement

The studies involving human participants were reviewed and approved by The Ethics Committee of the Institutional Review Board of the National Rehabilitation Center in South Korea. Written informed consent for participation was not required for this study in accordance with the national legislation and the institutional requirements.

## Author Contributions

JJL and J-HS contributed to conception and design of the study, performed the statistical analysis, and wrote the manuscript. All authors contributed to the article and approved the submitted version.

## Conflict of Interest

The authors declare that the research was conducted in the absence of any commercial or financial relationships that could be construed as a potential conflict of interest.
