# Supplementary material for: Predicting Clinically Significant Improvement After Robot-Assisted Upper Limb Rehabilitation in Subacute and Chronic Stroke
Source: Front Neurol. 2021 Jul 1;12:668923. doi: 10.3389/fneur.2021.668923 (PMC8281036; doi:10.3389/fneur.2021.668923)
Supplement: Supplementary file 1 [file Table_1.docx]

Supplementary Material

**Supplementary Table 1.** Univariable Analyses Using the MCID of the FMA-UE as the Outcome Measure According to Stroke Phase.

|  | Subacute phase | | | | Chronic phase | | | | |
| --- | --- | --- | --- | --- | --- | --- | --- | --- | --- |
| Baseline characteristics | Odds ratio | 95% CI | | *P-*value | Odds ratio | 95% CI | | *P*-value | |
| Demographic characteristic | | | | | | | | |  |
| Sex | 1.92 | 0.67, | 5.50 | 0.226* | 1.57 | 0.30, | 8.29 | 0.594 | |
| Age | 1.01 | 0.97, | 1.05 | 0.613 | 1.01 | 0.96, | 1.06 | 0.753 | |
| Time since onset | 0.99 | 0.97, | 1.00 | 0.046* | 0.99 | 0.98, | 1.00 | 0.043* | |
| Stroke subtype | 1.32 | 0.52, | 3.35 | 0.566 | 0.50 | 0.13, | 1.90 | 0.308 | |
| Stroke lesion | 0.72 | 0.29, | 1.81 | 0.484 | 0.53 | 0.13, | 2.19 | 0.385 | |
| Hemiplegic side | 1.71 | 0.67, | 4.38 | 0.261 | 0.78 | 0.22, | 2.82 | 0.704 | |
| Clinical measure | | | | | | | | |  |
| FMA‑A | 0.99 | 0.92, | 1.07 | 0.853 | 1.02 | 0.93, | 1.12 | 0.692 | |
| FMA‑B | 1.07 | 0.80, | 1.44 | 0.653 | 1.00 | 0.72, | 1.38 | 0.986 | |
| FMA‑C | 1.12 | 0.92, | 1.36 | 0.244* | 1.12 | 0.88, | 1.44 | 0.359 | |
| FMA‑D | 0.90 | 0.71, | 1.13 | 0.361 | 0.93 | 0.66, | 1.31 | 0.677 | |
| FMA‑Prox | 0.98 | 0.92, | 1.06 | 0.650 | 1.01 | 0.93, | 1.11 | 0.786 | |
| FMA‑Dist | 1.07 | 0.94, | 1.21 | 0.325 | 1.05 | 0.90, | 1.22 | 0.574 | |
| FMA‑UE | 1.00 | 0.95, | 1.06 | 0.965 | 1.02 | 0.95, | 1.08 | 0.664 | |
| MI‑pinch grasp | 1.02 | 0.96, | 1.08 | 0.462 | 1.04 | 0.96, | 1.14 | 0.325 | |
| MI‑elbow flexion | 1.01 | 0.92, | 1.20 | 0.904 | 1.04 | 0.93, | 1.17 | 0.453 | |
| MI‑shoulder abduction | 1.04 | 0.95, | 1.14 | 0.421 | 1.05 | 0.94, | 1.16 | 0.392 | |
| MI‑upper limb | 1.01 | 0.98, | 1.05 | 0.427 | 1.03 | 0.98, | 1.09 | 0.210* | |
| MRC‑shoulder flexion | 1.09 | 0.64, | 1.87 | 0.749 | 0.84 | 0.34, | 2.07 | 0.708 | |
| MRC‑shoulder extension | 1.06 | 0.62, | 1.78 | 0.842 | 1.03 | 0.44, | 2.43 | 0.940 | |
| MRC‑shoulder abduction | 1.16 | 0.67, | 2.00 | 0.597 | 0.92 | 0.39 | 2.19 | 0.857 | |
| MRC‑shoulder adduction | 1.03 | 0.59, | 1.80 | 0.926 | 1.20 | 0.52, | 2.77 | 0.677 | |
| MRC‑elbow flexion | 1.07 | 0.63, | 1.81 | 0.797 | 1.08 | 0.49, | 2.36 | 0.857 | |
| MRC‑elbow extension | 1.18 | 0.72, | 1.93 | 0.515 | 1.38 | 0.68, | 2.81 | 0.371 | |
| MRC‑wrist flexion | 1.45 | 0.85, | 2.45 | 0.170* | 1.24 | 0.55, | 2.81 | 0.600 | |
| MRC‑wrist extension | 1.65 | 0.94, | 2.90 | 0.081* | 1.75 | 0.81, | 3.79 | 0.155* | |
| MRC‑finger flexion | 1.20 | 0.73, | 1.95 | 0.474 | 1.59 | 0.84, | 3.02 | 0.155* | |
| MRC‑finger extension | 1.47 | 0.83, | 2.61 | 0.185* | 2.75 | 1.05, | 7.17 | 0.039* | |
| MRC‑shoulder | 1.02 | 0.89, | 1.18 | 0.767 | 1.00 | 0.80, | 1.25 | 0.991 | |
| MRC‑elbow | 1.07 | 0.82, | 1.38 | 0.637 | 1.13 | 0.76, | 1.66 | 0.554 | |
| MRC‑wrist | 1.25 | 0.95, | 1.66 | 0.113* | 1.25 | 0.82, | 1.89 | 0.297 | |
| MRC‑finger | 1.17 | 0.88, | 1.55 | 0.286 | 1.58 | 1.00, | 2.50 | 0.048* | |
| MRC‑UE | 1.03 | 0.96, | 1.09 | 0.397 | 1.05 | 0.94, | 1.18 | 0.352 | |
| MAS | 0.66 | 0.36, | 1.21 | 0.180* | 0.75 | 0.34, | 1.64 | 0.466 | |
| HMS | 1.54 | 1.00, | 2.37 | 0.049* | 1.79 | 1.04, | 3.09 | 0.037* | |
| BRS‑upper arm | 1.29 | 0.80, | 2.07 | 0.297 | 1.43 | 0.65, | 3.17 | 0.374 | |
| BRS‑hand | 1.44 | 0.94, | 2.22 | 0.097* | 1.58 | 0.83, | 3.00 | 0.166* | |
| Robotic kinematic measure | | | | | | | | |  |
| Smoothness | 2.59 | 0.00, | 2156.0 | 0.781 | 132.2 | 0.00, | 4591194.1 | 0.360 | |
| Reach error | 3.65 | 0.00, | 49966.3 | 0.790 | 0.88 | 0.00, | 145700.4 | 0.983 | |
| Path error | 13934.4 | 0.00, | 85424749033814 | 0.407 | 0.01 | 0.00, | 41175560696 | 0.755 | |
| Independence | 2.16 | 0.14, | 34.4 | 0.586 | 1.02 | 0.03, | 37.5 | 0.992 | |

MCID, minimal clinically important difference; FMA, Fugl-Meyer Assessment; UE, upper extremity; CI, confidence interval; FMA-A, Fugl-Meyer Assessment sub-score for the shoulder/elbow; FMA-B, Fugl-Meyer Assessment sub-score for the wrist; FMA-C, Fugl-Meyer Assessment sub-score for the hand; FMA-D, Fugl-Meyer Assessment sub-score for coordination; FMA-Prox, Fugl-Meyer Assessment sub-score for the proximal unit of the shoulder/elbow and coordination; FMA-Dist, Fugl-Meyer Assessment sub-score for the distal unit of the wrist and hand; MI, Motricity Index; MRC, Medical Research Council Scale for Muscle Strength; MAS, Modified Ashworth Scale; HMS, Hand Movement Scale; BRS, Brunnstrom Recovery Stage.

**P*<0.25 in the univariable analysis.
